# Salmon Calcitonin Attenuates Some Behavioural Responses to Nicotine in Male Mice

**DOI:** 10.3389/fphar.2021.685631

**Published:** 2021-06-21

**Authors:** Cajsa Aranäs, Jesper Vestlund, Sarah Witley, Christian E. Edvardsson, Aimilia Lydia Kalafateli, Elisabet Jerlhag

**Affiliations:** Department of Pharmacology, Institute of Neuroscience and Physiology, The Sahlgrenska Academy at the University of Gothenburg, Gothenburg, Sweden

**Keywords:** appetite-regulatory hormones, amylin receptors, calcitonin receptors, reward, pharmacological treatments

## Abstract

The behavioural responses to nicotine involve appetite-regulatory hormones; however, the effects of the anorexigenic hormone amylin on reward-related behaviours induced by nicotine remain to be established. Previous studies have shown that the amylinergic pathway regulates behavioural responses to alcohol, amphetamine and cocaine. Here, we evaluated the effects of salmon calcitonin (sCT), an amylin and calcitonin receptor (CTR) agonist, on nicotine-induced locomotor stimulation and sensitisation as well as dopamine release in the nucleus accumbens (NAc) shell. Moreover, we investigated the effects of sCT on the acquisition and expression of nicotine-induced reward in the conditioned place preference (CPP) paradigm. Finally, we performed Western Blot experiments in an attempt to identify the levels of the amylin receptor components CTRa, CTRb, and RAMP1 in reward-related areas of mice responding differently to repeated injections of sCT and nicotine in the locomotor sensitisation test. We found that sCT blocked nicotine’s stimulatory and dopamine-releasing effects and prevented its ability to cause locomotor sensitisation. On the other hand, sCT did not alter nicotine-induced acquisition and expression of CPP. Lastly, sCT-nicotine treated mice from the locomotor sensitisation experiment displayed higher levels of total CTR, i.e. CTRa and CTRb together, in the reward-processing laterodorsal tegmental area (LDTg) of the brain compared to mice treated with vehicle-nicotine. Overall, the present data reveal that activation of CTR or/and amylin receptors attenuates certain nicotine-induced behaviours in male mice, further contributing to the understanding of appetite-regulatory peptides in reward regulation.

## Introduction

Nicotine use contributes to disabilities and deaths worldwide (Carter et al., 2015). Although the available pharmacological treatments assist in reducing nicotine prevalence and thereby reduce nicotine’s negative effects on health, the efficacy of these is limited (Duaso and Duncan, 2012). Highlighting the neurobiological mechanisms influencing nicotine reward may help in the development of novel therapies for nicotine use cessation. There is therefore a substantial need to define the neurocircuits crucial for behavioural responses induced by nicotine. The latter include reward-related behaviours, which are modulated by appetite-regulatory hormones, such as ghrelin and glucagon-like peptide-1 (GLP-1) [for review, see (Jerlhag, 2019)], as numerous studies show. However, the effects of other appetite-regulatory hormones, like amylin, on the nicotine-induced reward-related behaviours remain to be established.

The pancreatic hormone amylin has a number of well-established physiological properties. For example, its various effects on glycaemic control led to the approval of amylinergic agents for the treatment of diabetes type 1 and 2 (Ryan et al., 2005). In addition, amylin decreases homeostatic and hedonic feeding [for review, see (Hartter et al., 1991; Hay, 2017; Boyle et al., 2018)]. These anorexigenic properties involve amylin receptors (AMYRs) within brain regions highly linked to reward regulation like the laterodorsal tegmental area (LDTg), ventral tegmental area (VTA), and nucleus accumbens (NAc) (Mietlicki-Baase et al., 2013; Baisley and Baldo, 2014; Mietlicki-Baase et al., 2015; Mietlicki-Baase et al., 2017; Reiner et al., 2017). Therefore, AMYR agonists are evaluated as obesity therapies, as they reduce body weight in both rodents and humans (Aronne et al., 2007; Lutz, 2012; Young, 2012).

More recent studies have established that the amylinergic pathway modulates reward processing. Indeed, activation of AMYRs reduces the acute rewarding properties of alcohol in male mice and decreases alcohol intake in various animal models of alcohol use disorder (Kalafateli A. L. et al., 2019; Kalafateli A. L. et al., 2019; Kalafateli et al., 2020a; Kalafateli et al., 2020c). The decrease of these alcohol-related behaviours involves AMYRs within the LDTg, VTA and NAc shell (Kalafateli et al., 2020b). Besides alcohol, activation of the amylin pathway reduces some behavioural responses to amphetamine and cocaine (Twery et al., 1986; Clementi et al., 1996; Kalafateli et al., 2021). The AMYR consists of a core calcitonin receptor (CTR), which exists in two isoforms (CTRa and CTRb), and one out of three receptor activity–modifying proteins (RAMP−1, −2, and −3), thus forming the AMYR1-3 subtypes (Bower and Hay, 2016). The AMYR1 provides further interest for reward-regulation, as high alcohol-consuming rats show higher expression of the RAMP1 gene in NAc shell compared to rats consuming low amounts of alcohol (Kalafateli A. L. et al., 2019).

We hypothesized that since activation of AMYRs or/and CTRs attenuates reward induced by alcohol, amphetamine and cocaine, similar mechanisms could modulate nicotine’s reward-related behaviours. In the present studies, salmon calcitonin (sCT), an agonist of both the AMYR and CTR (Christopoulos et al., 1999) was administered in a series of behavioural studies in male mice. Firstly, the ability of sCT to attenuate the nicotine-induced locomotor stimulation and locomotor sensitisation was investigated. Secondly, the influence of sCT on the enhanced dopamine release in the NAc shell following nicotine was explored. Thirdly, the effects of sCT on the acquisition and expression of nicotine-induced reward was evaluated in the conditioned place preference (CPP) paradigm. Finally, Western Blot experiments were conducted in order to identify the levels of CTRa, CTRb and RAMP1 in brain reward-related areas of mice in the locomotor sensitisation test.

## Material and Methods

### Animals

Male NMRI mice (8–12 weeks old and 25–35 g body weight; Charles River, Sulzfeld, Germany) were used. NMRI mice were selected as this strain has displayed robust behavioural responses to nicotine in our previous studies, as reflected by activation of the mesolimbic dopamine system (Jerlhag and Engel, 2011; Egecioglu et al., 2013). It should however be mentioned that other strains of mice, as well as rats, show similar behavioural responses to nicotine (Tzschentke, 1998; Jerlhag and Engel, 2011; Natarajan et al., 2011; Egecioglu et al., 2013; Ignatowska-Jankowska et al., 2013) and similar effects to sCT (Kalafateli et al., 2020b). The mice were group-housed and maintained at a 12/12-h light/dark cycle; they acclimatized to the animal facility (temperature of 20°C with 50% humidity) one week before experiments. They had *ad libitum* access to water and standard chow (Teklad Rodent Diet; Envigo, Madison, WI, United States) before and after each experiment. An independent set of age-matched mice was used in each behavioural experiment. For the Western Blot experiments, the mice brains from the repeated sCT-nicotine locomotor sensitisation test were used. All experiments were approved by the Swedish Ethical Committee on Animal Research in Gothenburg (207–2014; 195–2014; 1,457–2018; 3,348–2020).

### Drugs

Nicotine ditartrate (Nicotine, Sigma-Aldrich; Stockholm, Sweden) was diluted in vehicle solution (sodium hydroxide was added until pH = 7). The dose of nicotine (0.5 mg/kg, IP) was calculated from the salt form (i.e., 1.081 μmol/kg of nicotine ditartrate). The dose was selected as it activates the mesolimbic dopamine system, as seen by enhanced locomotion, accumbal dopamine release and CPP in male mice (Jerlhag and Engel, 2011; Egecioglu et al., 2013). A low dose of nicotine (0.125 mg/kg, IP) was used on Day 8 of the locomotor sensitisation experiment. This was done to establish that mice previously treated (Day 1–5) with nicotine show locomotor response to a low dose of nicotine, whereas vehicle-treated mice do not respond similarly. Nicotine was injected 15 min prior to behavioural testing. sCT (Tocris Bioscience, Bristol, United Kingdom) was diluted in vehicle (0.9%, NaCl solution) and was administered intraperitoneally (IP) at the dose of 5 μg/kg, 30 min prior to nicotine administration. This dose of sCT and the timeline of administration were chosen as they have been established necessary for the attenuation of alcohol-mediated behaviours in rodents (Kalafateli A. L. et al., 2019; Kalafateli et al., 2020a). All drugs and vehicle solution were injected at a volume of 10 ml/kg.

### Locomotor Activity

Six sound-attenuated, ventilated and dim lit (20 lux) open field boxes (420 × 420 × 200 mm; Open Field Activity System; Med Associates Inc.; Georgia, Vermont, United States) were used. In this setup, 15 × 15 infrared beams at the bottom of the floor, allow a computer-based system to register the distance travelled (cm per 5 min) of each mouse. Two different locomotor activity experiments (experiment 1 and 2) were conducted as previously described (Kalafateli et al., 2020b).

#### Experiment 1: Effects of acute sCT Administration on Nicotine-Induced Locomotor Stimulation

This experiment was designed to evaluate the effects of an acute sCT (5 μg/kg, IP) injection on the ability of nicotine (0.5 mg/kg, IP) to cause locomotor stimulation in male mice. The mice were allowed to habituate to the open field boxes for 60 min before sCT or equal volume of vehicle was injected. After 30 min, nicotine or equal volume of vehicle was administered. 15 min later, the cumulative 60-min locomotor activity was registered. The following treatment groups were included: vehicle-vehicle, vehicle-nicotine, sCT-vehicle and sCT-nicotine.

#### Experiment 2: Effects of repeated sCT and Nicotine Administration on Locomotor Sensitisation

Repeated injections of an addictive drug, like nicotine, enhances the locomotor activity response over time, i.e., locomotor sensitisation (Segal and Mandell, 1974). This locomotor sensitisation experiment was designed similar to previous studies (Jerlhag and Engel, 2011; Wellman et al., 2011; Clifford et al., 2012; Egecioglu et al., 2013; Kalafateli et al., 2020a; 2021). Throughout the entire protocol (Day 1–5 and Day 8), the mice were allowed to habituate to the open field boxes for 30 min prior to drug treatment. Firstly, we assessed the effects of repeated sCT administration on the ability of repeated nicotine to cause locomotor sensitisation for 5 days (Day 1–5). Each day, the mice were injected with sCT (5 μg/kg, IP) or vehicle; and 30 min later, nicotine (0.5 mg/kg, IP) or equal volume of vehicle was administered. 15 min later, the activity of each mouse was registered for 30 min. The following treatment groups were thus created for Day 1–5: vehicle-vehicle, vehicle-nicotine, sCT-vehicle and sCT-nicotine. The mice were then left untreated for two days (Day 6–7).

Secondly, we assessed the how mice co-injected with sCT-nicotine during Day 1–5, respond to a low dose of nicotine compared to mice treated with vehicle-nicotine during Day 1–5. On Day 8, all mice from the previous treatment groups were injected with a low dose of nicotine (0.125 mg/kg, IP). 15 min following the injection, the cumulative locomotor activity was registered for 30 min. In this experiment, the following treatment groups were created: Veh/Nic-Nic, sCT/Nic-Nic, Veh/Veh-Nic, and sCT/Veh-Nic.

### Tissue Isolation and Western Blot Experiments

Western Blot experiments were conducted in an attempt to identify the levels of the components of the AMYR1 i.e., the two isoforms of CTR, namely CTRa and CTRb, and RAMP1. In addition, the total levels of CTRa and CTRb, i.e. CTR(a + b), were analysed. Therefore, the brains from the mice of *Experiment 2* were used similarly to previous studies (Kalafateli et al., 2020a). Following the locomotor activity test on Day 8, the mice were briefly exposed to isoflurane (Isoflurane Baxter) and then decapitated. The whole brain was isolated, placed into plastic tubes and snap frozen in −80°C. On a later occasion, the LDTg, VTA and the NAc region (including the core and shell subregions) were punched out from frozen tissue. In detail, the brain was placed in a cold mouse brain matrix (Zivic instruments, Pittsburg, PA, United States) and was coronally sectioned in slices of appropriate thickness by consulting a mouse brain atlas (Franklin and Paxinos, 1997). The selected section was placed under a microscope on a cold glass plate (mix of regular ice and dry ice) to avoid tissue degradation. A tissue biopsy punch (Zivic instruments, Pittsburg, PA, United States) was used to isolate the aforementioned areas from both hemispheres.

These brain isolates were placed in homogenization buffer (PBS, 0.1% Triton X-100, a protease-inhibitor cocktail tablet and 5 mM EDTA) and then homogenized with an ultrasound sonicator (Sonifier Cell Disruptor B30, Branson Sonic Power Co. Danbury, CT, United States). The protein concentration was determined (bicinchoninic acid biochemical assay, Quick Start Bovine Serum Albumin kit; Bio-Rad, Hercules, CA, United States). 10 μg (NAc) or 12 μg (VTA or LDTg) of protein per sample was mixed with loading Buffer (2x Laemmli Sample Buffer containing β-Mercaptoethanol; Bio-Rad, Hercules, CA, United States). The samples, as well as a ScanLater Western Blot Protein Ladder™ (marker for molecular weight) were loaded on electrophoresis gels (Mini-PROTEAN® TGX™ Precast Gel; Bio-Rad). The gels were run at 20 V for 5 min and at 300 V for another 18 min in a Mini-PROTEAN® Tetra Vertical Electrophoresis Cell (Bio-Rad). For protein transferring, Trans-Blot® Turbo™ Mini PVDF (Bio-Rad) membranes were used and were run in a Trans-Blot® Turbo™ Transfer System (Bio-Rad). The membranes were blocked with custom made tris-Buffered Saline plus Tween® 20 (Merck Millipore, Burlington, MA, United States) containing 5% Blotting-Grade Blocker non-fat milk powder (Bio-Rad). The membranes were treated with dilutions of the primary and secondary antibodies (Table 1), and TBS-T was used as the washing agent between the steps. The dried membranes were later visualized in a SpectraMax i3v Platform (Molecular Devices, San Jose, CA, United States).

**TABLE 1:** Antibodies and dilutions used in the Western Blot experiments.

Target protein	Primary antibody (Abcam, Cambridge, United Kingdom)	Dilution/Incubation	Secondary antibody, (molecular devices, San Jose, CA, United States)	Dilution/Incubation
CTRa, CTRb, and CTR (a + b)	Anti-CTR (ab11042)	1:1,000 in TBS-T solution, overnight	Eu‐Labeled Goat Anti‐Rabbit ScanLater™	1:5,000 in TBS‐T, overnight
COXIV (reference protein)	Anti‐COXIV (ab14744)	1:5,000 in TBS‐T + 5% nonfat dry milk solution, overnight	Eu‐Labeled Goat Anti‐Mouse ScanLater™	1:5,000 in TBS‐T, overnight
RAMP1	Anti‐RAMP1 (ab156575)	1:1000 TBS‐T solution, overnight	Eu‐Labeled Goat Anti‐Rabbit ScanLater™	1:1,000 in TBS‐T, overnight
GAPDH (reference protein)	Anti‐GAPDH (ab8245)	1:10,000 in TBS‐T + 5% nonfat dry milk solution, overnight	Eu‐Labeled Goat Anti‐Mouse ScanLater™	1:10,000 in TBS‐T, overnight

CTR, calcitonin receptor; TBS‐T, Tris‐buffered saline; eu, europium; RAMP1, receptor activity modifying protein 1; COXIV, cytochrome c oxidase subunit IV; GAPDH, glyceraldehyde 3‐phosphate dehydrogenase.

### *In Vivo* Microdialysis Experiments

The microdialysis experiments in freely moving male mice were designed to assess the effects of an acute sCT (5 μg/kg, IP) injection on nicotine-induced (0.5 mg/kg, IP) dopamine release in the NAc shell. The selected dose of sCT, or vehicle, do not influence dopamine release in the NAc shell *per se* (Kalafateli A. L. et al., 2019), therefore this was not investigated in the present study.

A dialysis probe was surgically implanted two days prior to the microdialysis experiment. For the surgeries, the mice were anesthetized with isoflurane (Isoflurane Baxter; Univentor 400 Anaesthesia Unit, Univentor Ltd., Zejtun, Malta), placed in a stereotaxic frame (David Kopf Instruments; Tujunga, CA, United States) and kept on a heating pad to prevent hypothermia. The skull bone was exposed, one hole for the probe and one for the anchoring screw were drilled. Two drops of Xylocaine (10 mg/ml) adrenaline (5 μg/ml) (Aspen Nordic; Kronans Apotek, Gothenburg, Sweden) was used as local anaesthetic. NAc shell coordinates (Supplementary Figure S1) relative to bregma were used (Paxinos and Watson, 1998). The probe was alternated to either the left or the right side of the brain in a balanced setup. After surgery, the mice were injected with carprofen (5 mg/kg subcutaneous, Rimadyl®; Zoetis, Kronans Apotek, Gothenburg, Sweden) to relieve pain and were kept in individual cages (Macrolon III).

During the microdialysis experiment, the probe was connected to a microperfusion pump (U-864 Syringe Pump; AgnThós AB) and the mice were allowed to habituate to the microdialysis set-up for 60 min. The probe was perfused with Ringer solution (NaCl 140 mM, CaCl_2_ 1.2, KCl 3.0, and MgCl_2_ 1.0 mM [Merck KGaA Darmstadt, Germany)] at a rate of 1.6 μl/min and subsequently dialysate samples were collected in 20-min intervals across the entire test session (from −60 to 180 min). The baseline dopamine levels were defined as the average of two consecutive samples (−60 to −-40 min). At -30 min, sCT or an equal volume of vehicle was administered IP. Thirty minutes later (at 0 min), nicotine was injected IP to all mice and nine additional samples were collected. Collectively, the following two treatment groups were created: vehicle-nicotine and sCT-nicotine.

Dopamine was separated and quantified using two different high-performance liquid chromatography apparatuses with electrochemical detection as described previously (Kalafateli A. L. et al., 2019; Vallöf et al., 2019). After termination of the microdialysis experiments the mice were euthanized after exposure to, firstly, isoflurane (Isoflurane Baxter) and secondly CO_2_ and the endpoint was determined. Subsequently, the location of the probe was determined using the brain atlas by observation (Paxinos and Watson, 1998). The position of the active space of the microdialysis probe was determined for each animal tested. Only data from animals with placements within NAc shell was included in the statistical analysis (Supplementary Figure S1). This allows dopamine collection only in the NAc shell, rather than neighbouring regions.

### Conditioned Place Preference

By means of the CPP paradigm, the effects of sCT (5 μg/kg, IP) on either nicotine-induced (0.5 mg/kg, IP) reward (Test 1, acquisition of CPP) or reward-dependent memory retrieval of nicotine (Test 2, expression of CPP) were evaluated. The effect of vehicle or sCT on the acquisition or expression of CPP was not investigated herein as previous studies have demonstrated that sCT does not alter CPP *per se* (Kalafateli et al., 2020b). These controls were omitted to reduce the number of animals used. The CPP effect on nicotine *per se* has been established in previous studies (Tzschentke, 1998; Jerlhag and Engel, 2011; Natarajan et al., 2011; Egecioglu et al., 2013; Ignatowska-Jankowska et al., 2013). A biased design was used for both CPP experiments. Possible limitations with this model might reflect confounding rewarding and anxiolytic properties of an addictive drug. Moreover, novelty-seeking behaviours could possibly influence the outcome of the data (Tzschentke, 1998). Further, other studies with nicotine in rats have showed a strong CPP response following the biased (Brielmaier et al., 2008), but not the unbiased procedure (Calcagnetti and Schechter, 1994).

The CPP apparatus (24 × 50 × 24 cm; custom made at the University of Gothenburg, Sweden) consists of two compartments divided by a subtractable wall. The two compartments are defined by tactile and visual cues, where one compartment has white walls and white rough varnished floor, whereas the other has striped walls and a brown smooth varnished floor. All experiments were conducted in dim lit rooms, during the animal’s light phase. Each experiment consisted of three different phases: pre-conditioning (day 1), conditioning (days 2-5) and post-conditioning (day 6). During pre-conditioning, the mice were placed on the midline of the apparatus with free access to both compartments for 20 minutes. The mice preference for either side was subsequently recorded and the least preferred compartment was paired with drug administration i.e. biased procedure (Kalafateli et al., 2020b).

In Test 1 (acquisition of CPP), the mice were untreated during pre- and post-conditioning. During each of the four conditioning days, sCT or vehicle was injected 30 min prior to the nicotine or vehicle injection and 15 min later the mice were placed to the relevant compartment (20 min duration). The injections were altered between morning and afternoon in a balanced design, with an equal number of animals per group (*N* = 8 per group). On the post-conditioning day, the mice were placed on the midline of the apparatus with free access to both compartments for 20 min. The entire experiment was recorded with a video-camera allowing subsequent analysis of the time the mouse spent in each compartment.

In Test 2 (expression of CPP), the mice received a vehicle injection (IP) 30 min prior to the 20-min exposure to the apparatus during the pre-conditioning. On each of the four conditioning days, nicotine or vehicle was administered 15 min prior to the initiation of the experiment. On the post-conditioning day, sCT or vehicle was administered 30 min prior the exposure to the CPP apparatus. Then, the mice (*N* = 8 per group) were placed on the midline of the apparatus with free access to both compartments for 20 min.

### Data Analysis

The data from the locomotor activity *Experiment 1* were calculated as percentage from baseline and were analysed using two-way ANOVA (between subjects) for comparison between treatments.

The sensitisation data (Day 1–5) from the locomotor activity *Experiment 2* were analysed using two-way repeated measures ANOVA for comparison between treatments across time points for experimental Days 1–5. The difference in locomotor stimulation response (D5—D1) was analysed using one-way ANOVA. Tukey’s post-hoc test was used for multiple pairwise comparisons for all the locomotor activity experiments. The locomotor activity data from Day 8 were analysed with an un-paired *t*-test, as only some mice were pre-treated with nicotine during Day 1–5.

The microdialysis experiments analysis was conducted using two-way repeated measures ANOVA, followed by Tukey’s post-hoc test for multiple comparisons between treatments and across time points.

The Western Blot bands were quantified using the ImageJ software program (public domain software; NIH, MD, United States) and the data were analysed using the non-parametric Kruskal-Wallis *H* test on the normalized intensity ratios (Protein of interest (CTRa, CTRb, CTR (a + b)/COXIV or RAMP1/GAPDH). The Western Blot data are analysed as medians with interquartile range. Following significance, post-hoc analysis was done using the Dunn’s multiple comparisons test. Dunn’s post-hoc test was used as this is the appropriate procedure for multiple pairwise comparison following a non-parametric test. The non-parametric Mann-Whitney test was used for one sub-analysis of the Western Blot data and for the analysis comparing the normalized protein levels of CTRa and CTRb in each area (*p* < 0.025 significant given multiple testing). One-way ANOVA (between subjects) analysis was performed on the raw intensity data of COX-IV and GAPDH (absolute values which are normally distributed), to assess possible changes of the reference protein between the treatment groups.

In Test 1 and Test 2 CPP was calculated as the difference in % of the total time spent in the drug-paired compartment during the post-conditioning and pre-conditioning sessions: 

$$\frac{\left(\mathrm{Time in drug paired chamber postconditioning}-\mathrm{Time in drug paired chamber preconditioning}\right)}{1200}\times 100$$

The CPP data from Tests 1 and 2 were analysed with an un-paired t-test. In an attempt to analyse the ability of nicotine to cause a CPP in each test, a one sample t-test with a hypothetical mean of 0 was used to analyse the confidence interval in each treatment group.

Data are presented as mean ± SEM. For all experiments, a probability value of P < 0.05 was considered statistically significant. GraphPad Prism version 9, was used for all the statistical analysis.

## Results

### Acute sCT Administration Attenuates Nicotine-Induced Locomotor Stimulation and Dopamine Release in the NAc Shell in Male Mice

**FIGURE 1:**
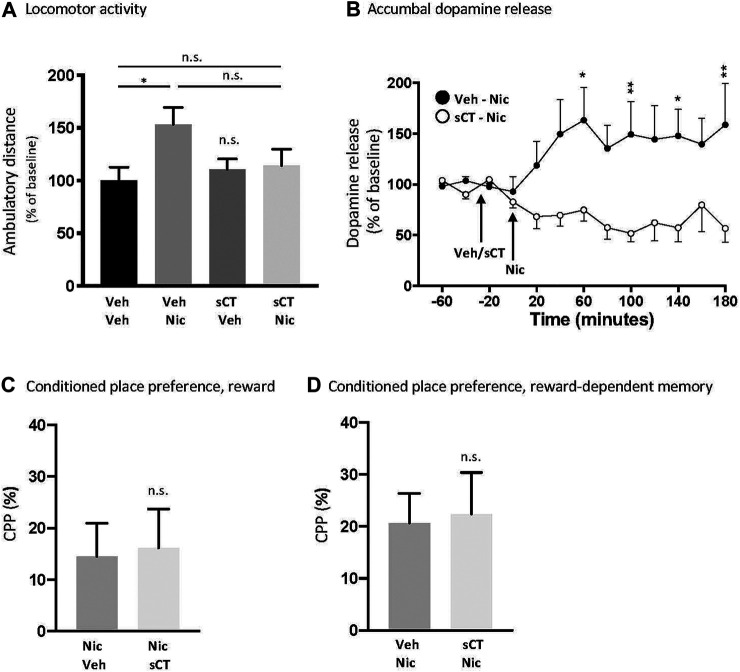
Effects of acute systemic sCT administration on nicotine-induced behaviours in male mice **(A)** Nicotine (Nic, 0.5 mg/kg, IP) caused locomotor stimulation in mice pre-treated with vehicle (Veh), but not in mice pre-treated with sCT (5 μg/kg, IP) (*p < 0.05 and n. s. not significant) **(B)** Dopamine release in the NAc shell induced by nicotine (0.5 mg/kg, IP) was higher in mice pre-treated with vehicle compared to those pre-treated with sCT (5 μg/kg, IP) at the time points of 60, 100, 140, and 180 min (*p < 0.05, **p < 0.01) **(C)** Nicotine-induced (0.5 mg/kg, IP) reward and **(D)** nicotine reward-dependent memory retrieval in the CPP paradigm was not affected by sCT (5 μg/kg, IP) compared to vehicle (Veh) (n. s. not significant). Data are presented as mean ± SEM.

There was an overall effect of treatment [F (1, 26) = 4.33, *p* = 0.0474], but not of pre-treatment (F (1, 26) = 1.09, *p* = 0.3070) or pre-treatment x nicotine interaction (F (1, 26) = 3.26, *p* = 0.0824) on locomotor stimulation in male mice (Figure 1A). Specifically, nicotine increased locomotor activity in mice (*p* = 0.0494, *N* = 8) when compared to vehicle (*N* = 7). When compared to the vehicle group, nicotine did not cause locomotor stimulation in sCT pre-treated mice (*p* = 0.8917, *N* = 7). There were no significant differences between the vehicle-nicotine and sCT-nicotine groups (*p* = 0.2087). sCT did not affect locomotor activity in mice when compared to vehicle (*p* = 0.9481, *N* = 8). sCT or nicotine administration did not affect any other behaviour measured during the 60 min spent in the open field boxes (Supplementary Figures S2A–E).

There was an overall effect of treatment [F (1,20) = 10.13, *p* = 0.0047] and time × treatment interaction [F (12,240) = 3.01, *p* = 0.0060], but not of time [F (12,240) = 0.48, *p* = 0.9273] on nicotine-induced dopamine release in the NAc shell in male mice (Figure 1B, *N* = 11 per group). Specifically, the nicotine-induced dopamine release was lower in mice pre-treated with sCT compared to those pre-treated with vehicle at the time points of 60 (*p* < 0.05), 100 (*p* < 0.01), 140 (*p* < 0.05), and 180 (*p* < 0.01) minutes.

### sCT Does Not Alter the Outcome in the CPP Tests in Male Mice

In the CPP paradigm, sCT did not attenuate nicotine-induced reward [Figure 1C, t (14) = 0.1687, *p* = 0.8684, *N* = 8 per treatment group] or memory retrieval of nicotine reward [Figure 1D, t (14) = 0.1798, *p* = 0.8599, *N* = 8 per treatment group] in male mice. A conditioning effect of nicotine were supported as nicotine tended to induce a place preference in the reward-CPP experiment (nicotine-vehicle *p* = 0.0601; nicotine-sCT *p* = 0.0695) and significantly induced a placed preference in the memory-dependent CPP experiment (vehicle-nicotine. *p* = 0.0084; sCT-nicotine *p* = 0.0261).

### sCT Prevents Nicotine-Induced Locomotor Sensitisation in Male Mice

**FIGURE 2:**
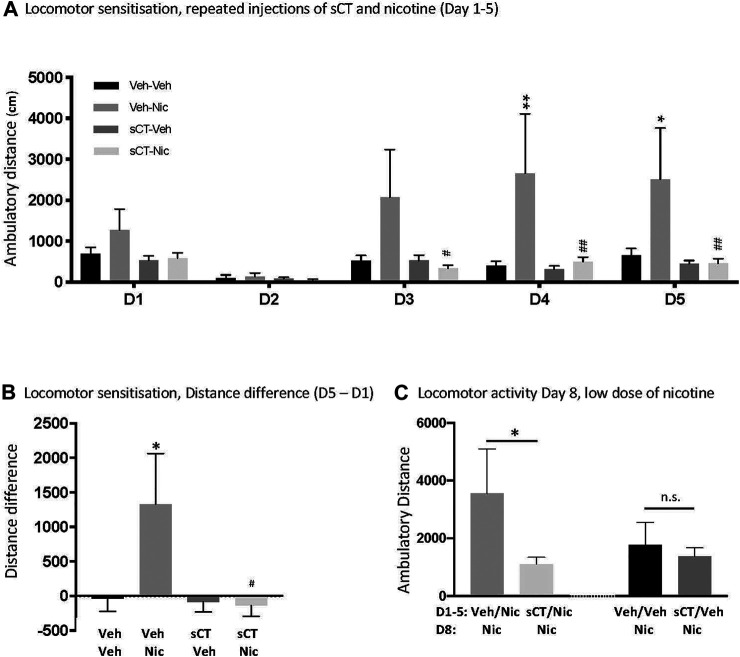
Effects of repeated administration of sCT and nicotine on locomotor sensitisation in male mice **(A)** Repeated nicotine (Nic) administration (0.5 mg/kg, IP) for 5 days (D1-D5) increased locomotion in mice, when compared to vehicle (Veh) at days 4 and 5 of administration (*p < 0.05, **p < 0.01, for the Veh-Nic *vs*. Veh-Veh comparisons). Locomotor stimulation was lower at Day 3–5 in mice pre-treated with sCT (5 μg/kg, IP) prior to nicotine, compared to those pre-treated with vehicle (^#^ *p* < 0.05, ^##^ *p* < 0.01, for the Veh-Nic *vs*. sCT-Nic comparisons). There was no difference in nicotine locomotor response between the sCT-nicotine or vehicle-vehicle treated mice. sCT had no effect *per se* on locomotor activity at any day, when compared to the vehicle group **(B)** The difference of travelled distance between D5-D1 was higher in vehicle-nicotine treated mice compared to vehicle-vehicle treated mice (*p < 0.05, for the Veh-Nic *vs*. Veh-Veh comparisons). The D5-D1 difference of travelled distance was lower in the sCT-nicotine compared to vehicle-nicotine treated mice (^#^ *p* < 0.05). There was no difference in locomotor response between vehicle-vehicle and sCT-nicotine treated mice **(C)** On Day 8 (D8), mice previously treated with vehicle-nicotine (Veh/Nic) during Day 1–5 (D1-5) showed higher locomotion response to a low dose of nicotine (Nic, 0.25 mg/kg, IP), compared to mice treated with sCT-nicotine (sCT/Nic) during D1-D5 (*p < 0.05). There was no difference in the locomotor activity response to a low dose of nicotine (Nic) between groups previously treated with vehicle-vehicle (Veh/Veh) or sCT-vehicle (sCT/Veh) during D1-D5. Data are presented as mean ± SEM.

There was no overall effect of treatment [F (3, 24) = 2.69, *p* = 0.0687], but an effect of time [F (4, 96) = 8.30, *p* < 0.0001] and time × treatment interaction [F (12, 96) = 2.92, *p* = 0.0017] on nicotine-induced locomotion in mice (Figure 2A). Post hoc analysis revealed no statistically significant differences between the experimental groups on Day 1 and 2 of the experiment (*p* > 0.05 for all). On Day 3, there were no differences (*p* = 0.1085) between the vehicle-vehicle (*N* = 6) and vehicle-nicotine (*N* = 7) groups. However, mice pre-treated with sCT (*N* = 7) had significantly decreased nicotine-induced locomotion (*p* = 0.0368) when compared to mice pre-treated with vehicle (*N* = 7). Compared to the vehicle-receiving group, nicotine administration increased locomotor activity in mice on Day 4 (*p* = 0.0068). On the same day, nicotine-induced locomotion was blocked in mice pre-treated with sCT when compared to the mice pre-treated with vehicle (*p* = 0.0054). Similarly, on Day 5, nicotine administration increased locomotion in mice pre-treated with vehicle when compared to the vehicle-vehicle group (*p* = 0.0385). Locomotor stimulation caused by nicotine was blocked in mice pre-treated with sCT when compared to mice pre-treated with vehicle (*p* = 0.0091). There were no differences noted between the sCT-vehicle (*N* = 7) and vehicle-vehicle receiving groups at any experimental day (*p* > 0.05 for all).

One-way ANOVA revealed an effect on the difference in distance travelled on Day 5—Day 1 (F (3, 24) = 1.89, *p* = 0.0218; Figure 2B). There was a difference in locomotor activity response to nicotine over time compared to vehicle-treated mice (*p* = 0.0301). Moreover, the response to repeated nicotine was lower in mice pre-treated with sCT compared to those pre-treated with vehicle (*p* = 0.0127).

On Day 8, mice previously (Day 1–5) treated with Veh/Nic show a higher locomotor stimulation to a low dose of nicotine, compared to mice treated with sCT/Nic during Day 1–5 [t (12) = 1.8181, *p* = 0.0471; Figure 2C]. There was no difference in the locomotor activity response to a low dose of nicotine between groups previously treated with Veh/Veh or sCT/Veh [t (12) = 0.5145, *p* = 0.6163; Figure 2C].

### Effects of Repeated sCT and Nicotine Administration on the Levels of CTRa, CTRb, CTR (a + b) and RAMP1 in Reward-Related Brain Areas in Male Mice From the Sensitisation Experiment

There was no effect of treatment on the levels of the reference protein COX-IV in the LDTg [F (3, 27) = 0.02, *p* = 0.9968; Supplementary Figure S3A], VTA [F (3, 25) = 0.57, *p* = 0.6379; Supplementary Figure S3B], or NAc [F (3, 25) = 0.28, *p* = 0.8411; Supplementary Figure S3C], in the mice from locomotor activity *Experiment 2*. There was no effect of treatment on the levels of the reference protein GAPDH in the LDTg [F (3, 24) = 0.17, *p* = 0.9165; Supplementary Figure S3D], VTA [F (3, 24) = 0.17, *p* = 0.9162; Supplementary Figure S3E], or NAc [F (3, 25) = 0.25, *p* = 0.8623; Supplementary Figure S3F] in these mice.

**FIGURE 3:**
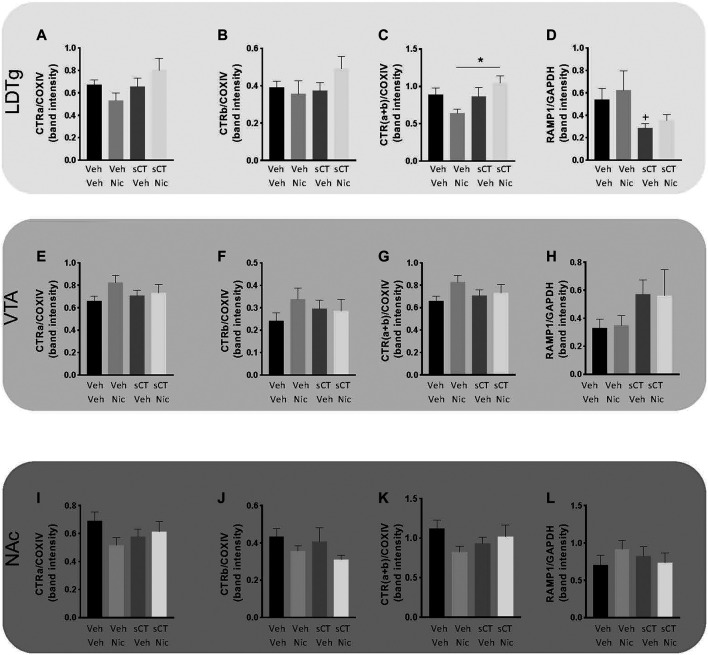
Effects of repeated sCT and nicotine administration on the levels of CTRa, CTRb, CTR (a + b) and RAMP1 in reward-related brain areas in male mice from the locomotor sensitisation experiment. In the laterodorsal tegmental area (LDTg) no effect of treatment was noted on the protein levels of normalized **(A)** CTRa or **(B)** CTRb **(C)** However, the protein levels of total CTR (a + b) were higher in the sCT-nicotine group compared to the vehicle-nicotine group (*p < 0.05) **(D)** No overall effect of treatment was noted on the protein levels of normalized RAMP1 in the LDTg. However, sub-analysis shows that sCT treatment reduces RAMP1 compared to vehicle (+*p* < 0.05, Mann-Whitney test). In the ventral tegmental area (VTA), no effect of treatment was noted on the protein levels of normalized **(E)** CTRa, **(F)** CTRb, **(G)** total CTR (a + b) or **(H)** RAMP1. In the nucleus accumbens (NAc), no effect of treatment was noted on the protein levels of normalized **(I)** CTRa, **(J)** CTRb, **(K)** total CTR (a + b) or **(L)** RAMP1. Data are presented as mean ± SEM.

In the LDTg (Figures 3A–D; Supplementary Figures S4A and B), no effect of treatment was noted on the normalized CTRa (*p* = 0.0888; mean ranks for vehicle-vehicle: 17.75, *N* = 8; vehicle-nicotine: 9.00, *N* = 7; sCT-vehicle: 15.75, *N* = 8 and sCT-nicotine: 20.63, *N* = 8; Figure 3A). Similarly, there was no effect of treatment on the normalized CTRb levels (*p* = 0.3036; mean ranks for vehicle-vehicle: 16.88, *N* = 8; vehicle-nicotine: 11.86, *N* = 7; sCT-vehicle: 14.38, *N* = 8 and sCT-nicotine: 20.38, *N* = 8; Figure 3B). In the same area, there was an overall effect of treatment on the total CTR (a + b) protein levels (*p* = 0.0184; Figure 3C) normalized to COXIV. Further analysis showed that this ratio was significantly higher in the sCT-nicotine group (mean rank: 22.38, *N* = 8) compared to the vehicle-nicotine group (mean rank: 7.57, *N* = 7) (*p* = 0.0099). There were no significant differences noted on the total levels of CTR between the other groups (mean ranks for vehicle-vehicle: 16.88, *N* = 8; sCT-vehicle: 16.13, *N* = 8). Moreover, no effect of treatment was noted on the normalized RAMP1 (*p* = 0.0646; mean ranks for vehicle-vehicle: 18.57, *N* = 7; vehicle-nicotine: 18.43, *N* = 7; sCT-vehicle: 9.00, *N* = 8 and sCT-nicotine: 12.50, *N* = 6; Figure 3D). In an attempt to replicate previous studies showing that sCT treatment reduces expression of RAMP1, a sub-analysis of the vehicle-vehicle group and the sCT-vehicle group was conducted for this group. This revealed a trend towards reduction in RAMP1 levels in the sCT compared to the vehicle-treated mice (*p* = 0.0205, Mann-Whitney test).

In the VTA (Figures 3E–H; Supplementary Figures S4C and D), there was no effect of treatment on the normalized CTRa (*p* = 0.1678; mean ranks for vehicle-vehicle: 10.29, *N* = 7; vehicle-nicotine: 20.43, *N* = 7; sCT-vehicle: 14.25, *N* = 8 and sCT-nicotine: 15.14, *N* = 8; Figure 3E), CTRb (*p* = 0.5488; mean ranks for vehicle-vehicle: 11.43; vehicle-nicotine: 18.00; sCT-vehicle: 15.25; sCT-nicotine: 15.29; Figure 3F) or CTR (a + b) levels (*p* = 0.1633; mean ranks for vehicle-vehicle: 10.00; vehicle-nicotine: 15.00; sCT-vehicle: 20.29; sCT-nicotine: 14.71; Figure 3G). No effect of treatment was noted on the normalized RAMP1 (*p* = 0.3725; mean ranks for vehicle-vehicle: 11.38, *N* = 8; vehicle-nicotine: 12.67, *N* = 6; sCT-vehicle: 18.13, *N* = 8 and sCT-nicotine: 15.65, *N* = 6; Figure 3H).

Lastly, in the NAc (Figures 3I–L; Supplementary Figures 4E and F), no effect of treatment was noted on the levels of CTRa (*p* = 0.3364; mean ranks for vehicle-vehicle: 18.00, *N* = 8; vehicle-nicotine: 11.00, *N* = 7; sCT-vehicle: 14.25, *N* = 8; sCT-nicotine: 15.33, *N* = 6; Figure 3I), CTRb (*p* = 0.3110; mean ranks for vehicle-vehicle: 18.75; vehicle-nicotine: 10.43; sCT-vehicle: 15.25; sCT-nicotine: 15.00; Figure 3J) and CTRa + b levels (*p* = 0.2940; mean ranks for vehicle-vehicle: 19.25; vehicle-nicotine: 14.25; sCT-vehicle: 10.86; sCT-nicotine: 15.17; Figure 3K). Furthermore, no effect of treatment was noted on the normalized RAMP1 (*p* = 0.5963; mean ranks for vehicle-vehicle: 12.00, *N* = 8; vehicle-nicotine: 17.86, *N* = 7; sCT-vehicle: 15.88, *N* = 8 and sCT-nicotine: 14.50, *N* = 6; Figure 3L).

Further Mann-Whitney analysis demonstrated that the protein levels of normalized CTRa isoform, rather than the CTRb, is the most profound in the LDTg (*p* = 0.0006, Supplementary Figure S5A) VTA (*p* = 0.0006, Supplementary Figure S5B), and NAc (*p* = 0.0070, Supplementary Figure S5C).

## Discussion

Overall, the present data revealed that activation of CTRs or/and AMYRs reduces certain nicotine-induced behaviours in male mice. Although sCT attenuated the nicotine-induced hypermotion, locomotor sensitisation and dopamine elevation in NAc shell, it did not alter the behavioural outcome to nicotine in the CPP paradigms. sCT-nicotine treated mice from the sensitisation experiment displayed higher levels of the total CTR (a + b) in the LDTg compared to the mice treated with vehicle-nicotine.

Similar to the present data, sCT blocks acute behavioural responses to alcohol in male mice (Kalafateli A. L. et al., 2019; Kalafateli A. L. et al., 2019; Kalafateli et al., 2020a; Kalafateli et al., 2020c). Moreover, sCT, or a selective AMYR agonist, reduce alcohol intake in various models of alcohol use disorder in male and female rats (Kalafateli A. L. et al., 2019; Kalafateli A. L. et al., 2019; Kalafateli et al., 2020a; Kalafateli et al., 2020c). Similarly, sCT attenuates the cocaine-induced locomotor stimulation and dopamine release in Nac shell (Kalafateli et al., 2021), and decreases the amphetamine-induced locomotor simulation (Twery et al., 1986; Clementi et al., 1996). In humans, cocaine reduces amylin in plasma (Bouhlal et al., 2017; Angarita et al., 2021). Additionally to reward induced by addictive drugs, the amylinergic pathway regulates natural rewards like sexual behaviours (Clementi et al., 1999) and hedonic feeding (Mietlicki-Baase et al., 2013; Baisley and Baldo, 2014; Mietlicki-Baase et al., 2015; Mietlicki-Baase et al., 2017; Reiner et al., 2017). This may be associated with the ability of sCT to prevent a reward from activating the mesolimbic dopamine system, as sCT prevents the VTA-stimulated or drug-induced dopamine release in Nac shell (Whiting et al., 2017; Kalafateli A. L. et al., 2019; Kalafateli et al., 2021)

We further found that sCT prevents the acquisition of nicotine-induced locomotor sensitisation when sCT and nicotine are co-administered repeatedly. This is evident as the enhanced locomotor activity caused by nicotine during Day 3–5 is not present in mice pre-treated with sCT prior to nicotine. Moreover, the activity is similar between vehicle and sCT-nicotine treated mice. Similarly, following repeated nicotine administration the difference in locomotor activity at Day 5 compared to Day 1 is higher in vehicle compared to sCT pre-treated mice. When exposed to a low dose of nicotine Day 8, mice previously treated with vehicle-nicotine display a higher locomotor activity compared to sCT-nicotine mice. In this sensitisation design, sCT is not present on the last experimental day, supporting that treatment with sCT during acquisition prevents the expression of nicotine-induced locomotor sensitisation following treatment termination. Similarly, mice previously treated with sCT display lower behavioural responses to alcohol (Kalafateli et al., 2020a) and lower intake of Ensure® diet (Boyle et al., 2018) compared to those previously treated with vehicle. Our current nicotine-induced sensitisation findings are in line with those showing that mice repeatedly co-treated with sCT-cocaine display lower locomotor response compared to those treated with vehicle-cocaine in the sensitisation setup (Kalafateli et al., 2021). Moreover, sCT initially reduces alcohol-induced locomotor response in mice co-treated with sCT and alcohol (Kalafateli et al., 2020a). Similar results have been obtained with other appetite-regulatory peptides. Indeed, a ghrelin receptor antagonist, or a GLP-1 receptor agonist, prevented nicotine-induced locomotor sensitisation in male rodents (Jerlhag and Engel, 2011; Wellman et al., 2011; Egecioglu et al., 2013). As locomotor sensitisation reflects neurobiological alterations important for craving and compulsive drug taking (Robinson and Berridge, 1993), the effect of sCT on nicotine self-administration in chronic nicotine models in rodents should be investigated. Such studies are also warranted in the future, in order to determine the impact of sCT on nicotine’s rewarding and motivational properties. Interestingly, a GLP-1 receptor agonist reduces nicotine self-administration in mice (Tuesta et al., 2017). This also raises the need for future studies exploring the potential association between amylin and nicotine craving in humans. Supportively, higher ghrelin levels or lower peptide YY levels are associated with higher nicotine craving in abstinent smokers (al'Absi et al., 2014; Koopmann et al., 2015).

The lower nicotine sensitisation in sCT-treated mice might involve the total CTR, for which sCT has the highest binding affinity (Christopoulos et al., 1999). Indeed, the total levels of CTR (a + b) in the LDTg are higher in sCT-nicotine compared to vehicle-nicotine treated mice from the sensitisation experiment. These Western Blot data may thus indicate that AMYR within the LDTg contribute towards the ability of sCT to attenuate nicotine-induced locomotor sensitisation. Supportively, local infusion of sCT into the LDTg reduces various alcohol-related behaviours (Kalafateli et al., 2021), attenuates locomotor stimulation caused by cocaine (Kalafateli et al., 2021) and reduces hedonic feeding behaviours in male rats (Reiner et al., 2017). It is therefore plausible that sCT, by increasing the CTR protein content possibly on GABAergic neurons (Reiner et al., 2017) in the LDTg, prevents the enhanced dopaminergic tone caused by nicotine though inhibition of the direct or indirect projections from the LDTg to NAc shell (Omelchenko and Sesack, 2005; Lammel et al., 2012). Whether this mechanism is physiologically relevant is currently unknown and relevant studies are thus warranted for the future. Although the present Western Blot experiments do not reveal any differences in any other investigated brain region, additional brain areas or peripheral organs where AMYR are expressed might participate in this sCT-nicotine interaction. A separate sub-analysis of the Western Blot data shows a trend towards lower protein levels of RAMP1 in the LDTg in mice treated with sCT compared to those treated with vehicle. Similarly, amylin administration reduces the mRNA levels of RAMP1 in area postrema (Liberini et al., 2016). The presence of the RAMP1 protein within the LDTg, VTA and NAc further strengthens the physiological role of RAMP1 within these reward areas where the expression of the RAMP1 gene has previously been detected (Sexton et al., 1994; Mietlicki-Baase et al., 2017; Reiner et al., 2017). A physiological role of RAMP1 is further supported as the expression of the RAMP1 gene in NAc shell is high compared to low-alcohol consuming rats (Kalafateli A. L. et al., 2019). The protein levels of RAMP2 or RAMP3 were not investigated herein due to lack of selective antibodies towards these targets. Therefore, the possibility that these subtypes modulate the behavioural responses caused by sCT cannot be excluded. Our Western Blot experiment further reveals that the CTRa isoform, rather than the CTRb, is the most profound in the three reward-related areas investigated. Relevant results have been obtained from rat studies in the genetic level, where the expression of the CTRa gene is higher over the CTRb gene in the LDTg (Liberini et al., 2016; Reiner et al., 2017; Kalafateli A. L. et al., 2019).

Although the present study presents a clear link between nicotine-induced behaviours and activation of CTRs or/and AMYRs, it is associated with certain limitations. Herein, only male mice were included; therefore, studies including females, which may respond differently to sCT, should be conducted in the future. It should also be considered that CTRs, rather than AMYRs are important for sCT’s effect on nicotine behaviours, as sCT is a potent CTR agonist. Studies investigating the effect of a selective AMYR agonist showed reduced alcohol intake (Kalafateli et al., 2020c) and similar studies on the effects of those selective AMYR agonists on the stimulatory properties of nicotine are also needed. Moreover, studies using AMYR antagonists would add further insight on the endogenous amylin pathway and how it modulates nicotine-related behaviours. The lack of data using a selective AMYR agonists and antagonists could be considered as limitation in the present study. In the present studies, sCT and nicotine were administered systemically, suggesting that potential peripheral effects might mediate the current findings. However, sCT has been detected in various brain areas following systemic administration (Zakariassen et al., 2020), including areas regulating reward (Kalafateli et al., 2020b). In the present study, sCT did not attenuate nicotine’s rewarding properties or reward-dependent memory retrieval in the CPP paradigm. Although sCT attenuates alcohol-induced CPP (Kalafateli A. L. et al., 2019), it does not affect CPP induced by cocaine in the same paradigm (Kalafateli et al., 2021). The discrepancy between the effect of sCT on nicotine-, cocaine- and alcohol-induced CPP, possibly indicates that different neurochemical systems modulate CPP induced by different addictive drugs. Additional CPP studies, defining such circuits, will be thus informative. We hypothesise that sCT reduces nicotine-induced locomotor stimulation, dopamine release in Nac shell and locomotor sensitisation, by preventing the interaction of nicotine with the mesolimbic dopamine system. However, the ability of sCT to attenuate behavioural responses to nicotine may involve other pathways, such as reduced stress or anxiety-like behaviours (Roth et al., 2009). Indeed, amylin protects from stress-induced hyperphagia in socially stressed rats (Smeltzer et al., 2012); given that social stress increases nicotine craving and puts smokers at risk of relapse (Buchmann et al., 2010; Watson et al., 2018), there is the speculation that amylin may protect from stress-induced nicotine relapse. Likewise, it was suggested that a GLP-1 receptor agonist decreases nicotine intake by increasing avoidance to nicotine rather than decreasing nicotine reward (Tuesta et al., 2017). Tentative research directives for upcoming studies.

The present data offer further insight into the association between activation of CTRs or/and AMYRs and reward-related behaviours. Indeed, sCT attenuates the ability of nicotine to cause hyperlocomotion, locomotor sensitisation and dopamine release in the NAc. Based on the current findings, clinical studies designed to evaluate the effect of AMYR agonists approved for diabetes type 1 and 2, like pramlintide (Ryan et al., 2005), on nicotine use are of substantial interest for the future.

## Data Availability

The raw data supporting the conclusions of this article will be made available by the authors, without undue reservation.
