# Supplementary material for: Salmon Calcitonin Attenuates Some Behavioural Responses to Nicotine in Male Mice
Source: Front Pharmacol. 2021 Jun 21;12:685631. doi: 10.3389/fphar.2021.685631 (PMC8257032; doi:10.3389/fphar.2021.685631)
Supplement: Supplementary file 1 [file Presentation1.PPTX]

## Slide 1
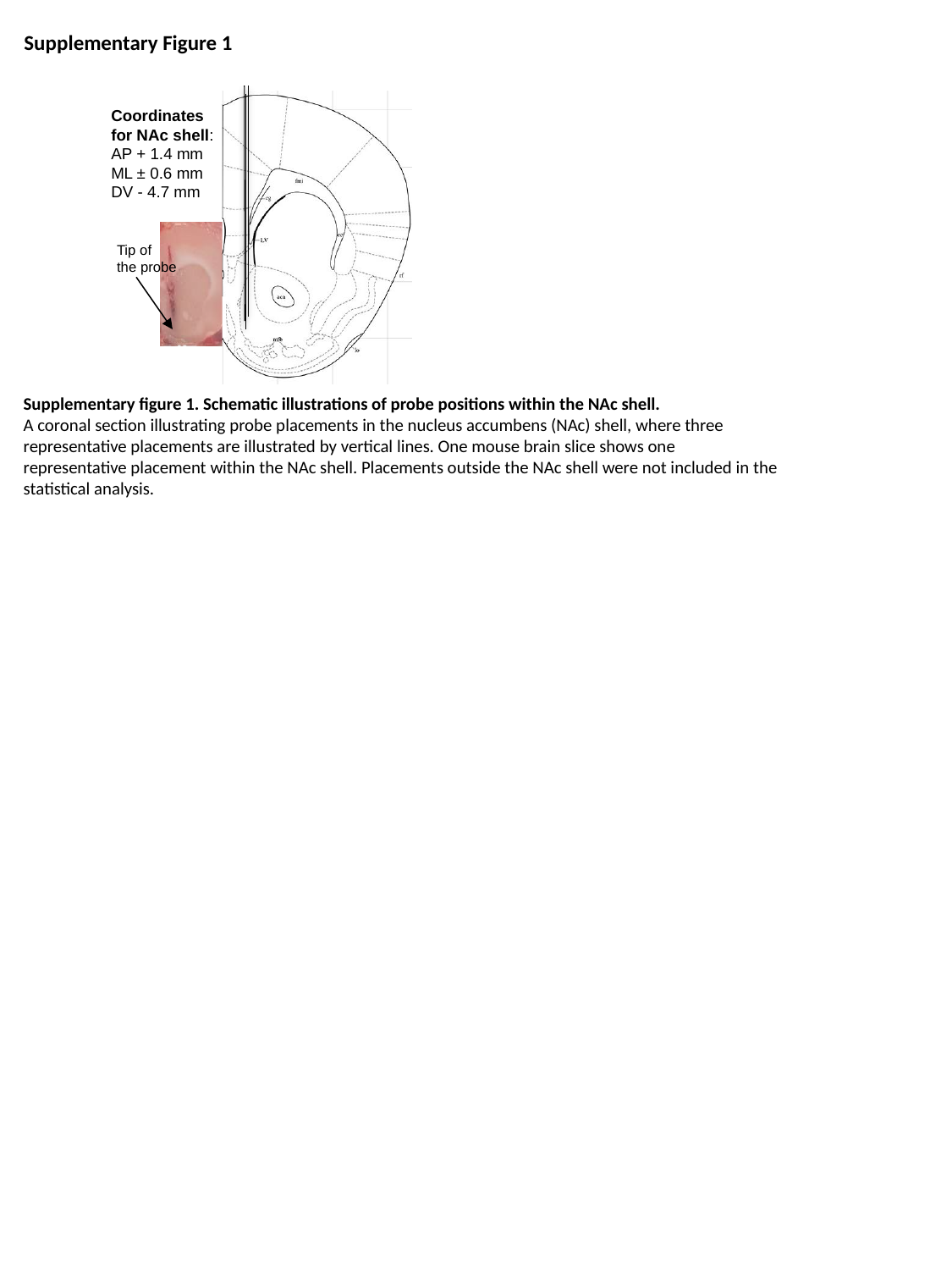

Supplementary Figure 1
Coordinates
for NAc shell:
AP + 1.4 mm
ML ± 0.6 mm
DV - 4.7 mm
Tip of
the probe
Supplementary figure 1. Schematic illustrations of probe positions within the NAc shell.
A coronal section illustrating probe placements in the nucleus accumbens (NAc) shell, where three representative placements are illustrated by vertical lines. One mouse brain slice shows one representative placement within the NAc shell. Placements outside the NAc shell were not included in the statistical analysis.

## Slide 2
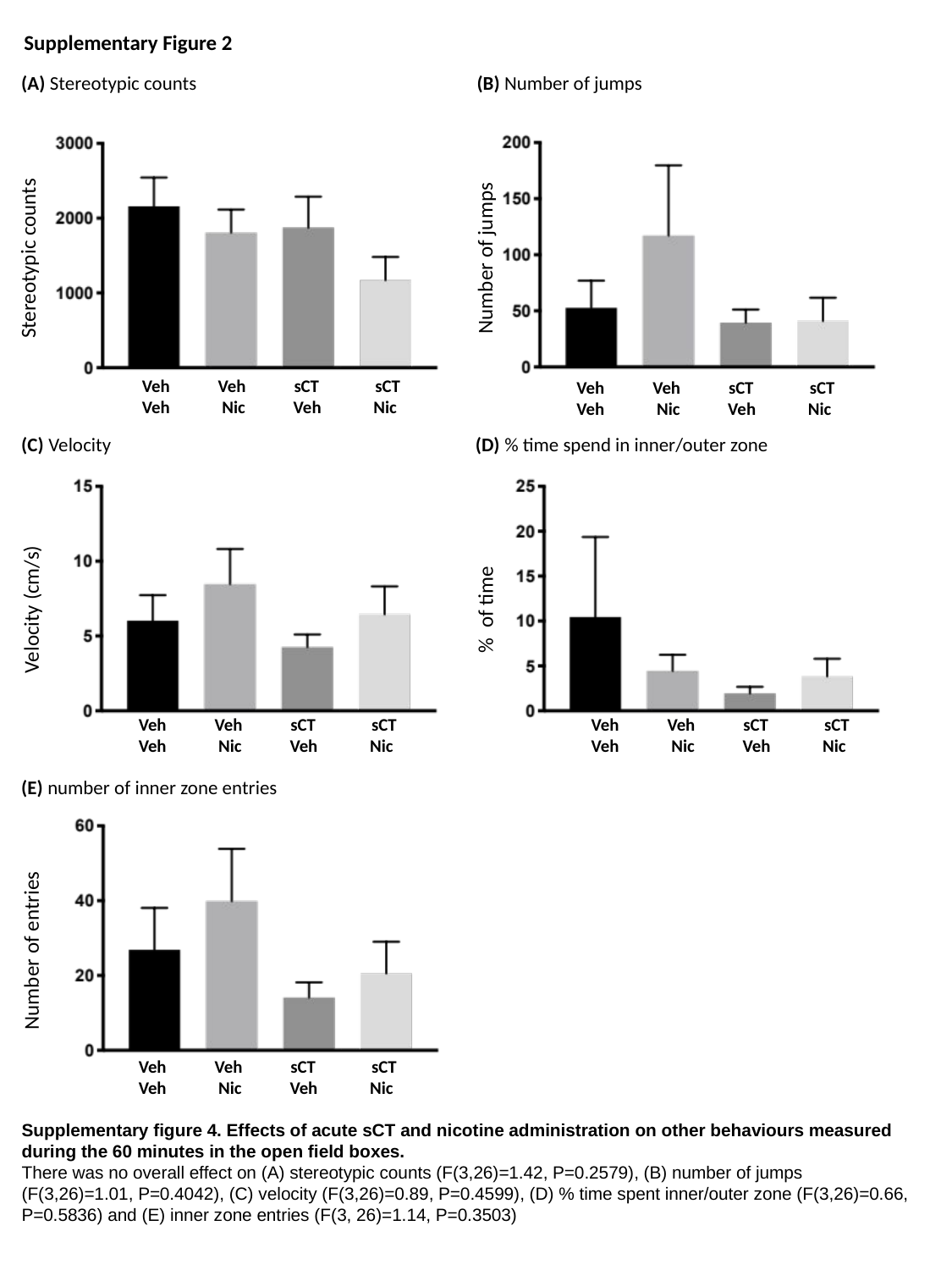

(A) Stereotypic counts (B) Number of jumps
Supplementary Figure 2
Stereotypic counts
Number of jumps
 Veh Veh sCT sCT
 Veh Nic Veh Nic
 Veh Veh sCT sCT
 Veh Nic Veh Nic
(C) Velocity (D) % time spend in inner/outer zone
Velocity (cm/s)
% of time
 Veh Veh sCT sCT
 Veh Nic Veh Nic
 Veh Veh sCT sCT
 Veh Nic Veh Nic
(E) number of inner zone entries
Number of entries
 Veh Veh sCT sCT
 Veh Nic Veh Nic
Supplementary figure 4. Effects of acute sCT and nicotine administration on other behaviours measured during the 60 minutes in the open field boxes.
There was no overall effect on (A) stereotypic counts (F(3,26)=1.42, P=0.2579), (B) number of jumps (F(3,26)=1.01, P=0.4042), (C) velocity (F(3,26)=0.89, P=0.4599), (D) % time spent inner/outer zone (F(3,26)=0.66, P=0.5836) and (E) inner zone entries (F(3, 26)=1.14, P=0.3503)

## Slide 3
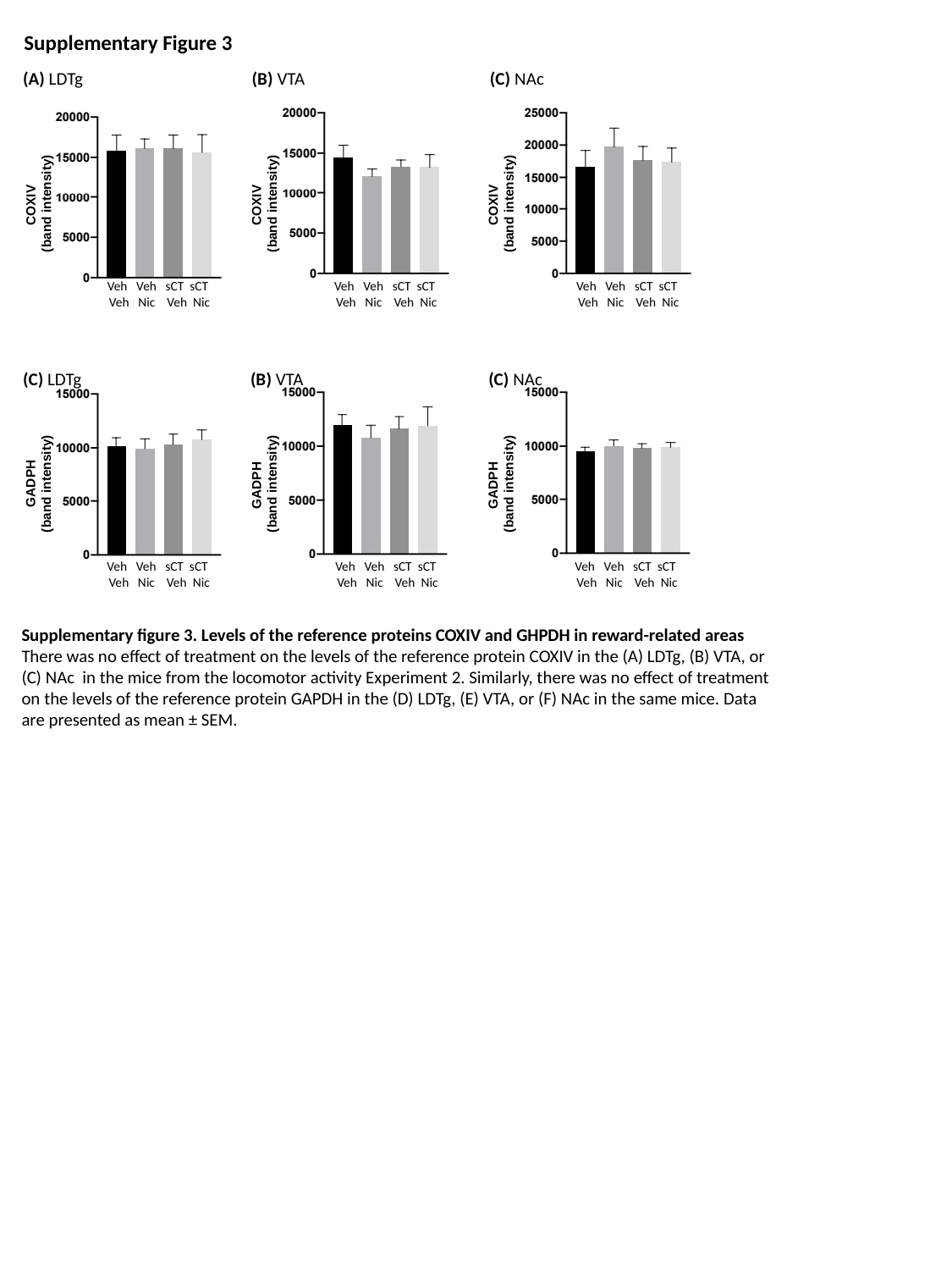

Supplementary Figure 3
(A) LDTg (B) VTA (C) NAc
(C) LDTg (B) VTA (C) NAc
COXIV
(band intensity)
COXIV
(band intensity)
COXIV
(band intensity)
 Veh Veh sCT sCT
 Veh Nic Veh Nic
 Veh Veh sCT sCT
 Veh Nic Veh Nic
 Veh Veh sCT sCT
 Veh Nic Veh Nic
GADPH
(band intensity)
GADPH
(band intensity)
GADPH
(band intensity)
 Veh Veh sCT sCT
 Veh Nic Veh Nic
 Veh Veh sCT sCT
 Veh Nic Veh Nic
 Veh Veh sCT sCT
 Veh Nic Veh Nic
Supplementary figure 3. Levels of the reference proteins COXIV and GHPDH in reward-related areas
There was no effect of treatment on the levels of the reference protein COXIV in the (A) LDTg, (B) VTA, or (C) NAc in the mice from the locomotor activity Experiment 2. Similarly, there was no effect of treatment on the levels of the reference protein GAPDH in the (D) LDTg, (E) VTA, or (F) NAc in the same mice. Data are presented as mean ± SEM.

## Slide 4
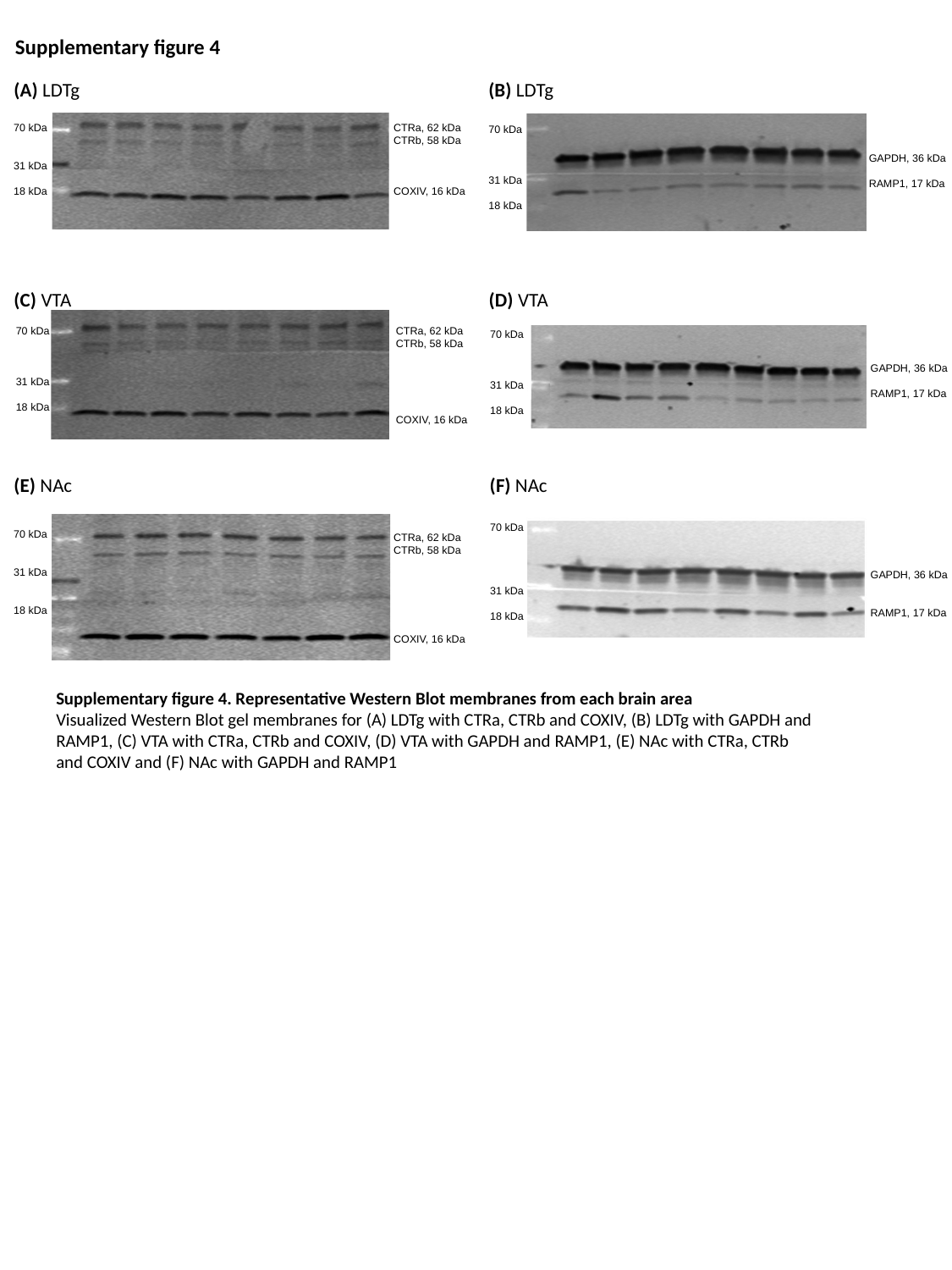

Supplementary figure 4
 (A) LDTg (B) LDTg
 (C) VTA (D) VTA
 (E) NAc (F) NAc
CTRa, 62 kDa
CTRb, 58 kDa
COXIV, 16 kDa
70 kDa
31 kDa
18 kDa
70 kDa
31 kDa
18 kDa
GAPDH, 36 kDa
RAMP1, 17 kDa
CTRa, 62 kDa
CTRb, 58 kDa
COXIV, 16 kDa
70 kDa
31 kDa
18 kDa
70 kDa
31 kDa
18 kDa
GAPDH, 36 kDa
RAMP1, 17 kDa
70 kDa
31 kDa
18 kDa
GAPDH, 36 kDa
RAMP1, 17 kDa
70 kDa
31 kDa
18 kDa
CTRa, 62 kDa
CTRb, 58 kDa
COXIV, 16 kDa
Supplementary figure 4. Representative Western Blot membranes from each brain area
Visualized Western Blot gel membranes for (A) LDTg with CTRa, CTRb and COXIV, (B) LDTg with GAPDH and RAMP1, (C) VTA with CTRa, CTRb and COXIV, (D) VTA with GAPDH and RAMP1, (E) NAc with CTRa, CTRb and COXIV and (F) NAc with GAPDH and RAMP1

## Slide 5
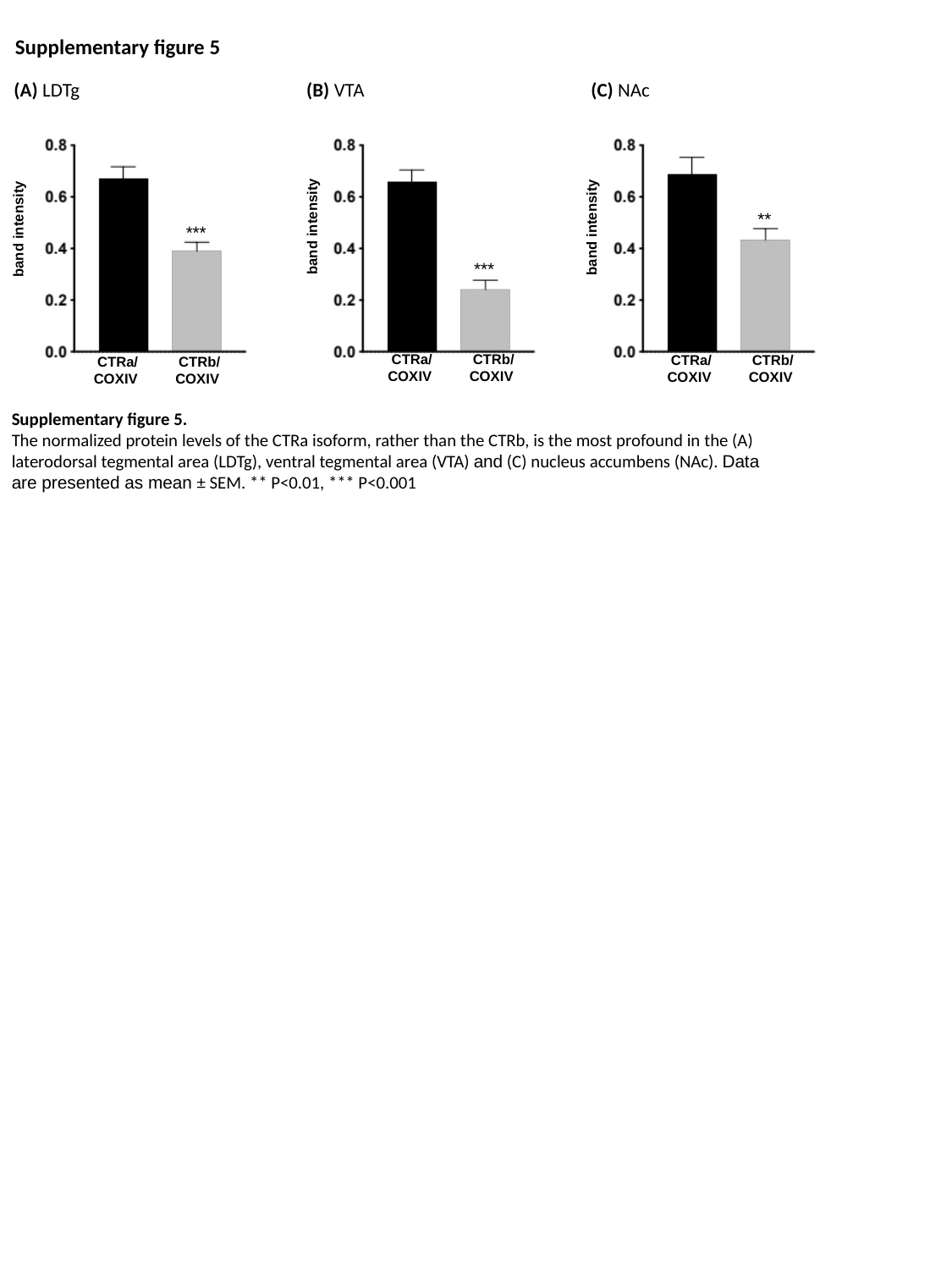

Supplementary figure 5
 (A) LDTg (B) VTA (C) NAc
**
band intensity
band intensity
band intensity
***
***
CTRa/
COXIV
CTRb/
COXIV
CTRa/
COXIV
CTRb/
COXIV
CTRa/
COXIV
CTRb/
COXIV
Supplementary figure 5.
The normalized protein levels of the CTRa isoform, rather than the CTRb, is the most profound in the (A) laterodorsal tegmental area (LDTg), ventral tegmental area (VTA) and (C) nucleus accumbens (NAc). Data are presented as mean ± SEM. ** P<0.01, *** P<0.001
